# A novel one-step multiplex PCR method (OS-MPCR) for efficient NGS library preparation in cancer

**DOI:** 10.1371/journal.pone.0357776

**Published:** 2026-09-15

**Authors:** Haiyan Feng, Xiao Hu, Lei Zhang, Qi Zhang, Shuocun Wang, Bingqing Lv, Yufeng Zhang, Dong Shi, Ping Zhou

**Affiliations:** 1 Department of Pathology, Zibo First Hospital, Zibo, Shandong, China; 2 Shanghai Yijian Health Technology Co., Ltd., Shanghai, China; ShanghaiTech University, CHINA

## Abstract

**Background:**

Next-generation sequencing (NGS) is essential for precision oncology, yet its clinical implementation is often hindered by limited sample quantity, complex workflows, and cross-contamination risks. We evaluated a modified One-Step Multiplex PCR (OS-MPCR) assay designed to streamline library construction while maintaining high analytical sensitivity and diagnostic accuracy.

**Methods:**

The OS-MPCR method integrates adapter ligation and library amplification into a single, closed-tube reaction, eliminating intermediate open-tube manipulations. The analytical performance was evaluated using standard reference materials targeting SNVs, Indels, and fusions. Clinical validation was performed on retrospective formalin-fixed paraffin-embedded (FFPE) specimens (DNA, N = 76; RNA, N = 34) from lung cancer patients, using well-established liquid-phase hybrid capture assays as the clinical verification standard.

**Results:**

OS-MPCR streamlined the workflow, reducing hands-on time to just 20 minutes. The assay enabled reliable variant detection at 1% frequency with as little as 1 ng of DNA for SNVs/Indels and 5 ng of total RNA for fusions, with low-input quantification limits inherently bounded by absolute molecular copy numbers. In the clinical cohort, OS-MPCR demonstrated 100% diagnostic concordance with the verification method. Quantitative analysis revealed a strong correlation in variant allele frequencies (Spearman *r* = 0.9777, 95% CI: 0.9668–0.9851, *P* < 0.0001), with libraries consistently achieving highly reproducible yields and stable, near 1.0 target rates across the entire cohort.

**Conclusion:**

OS-MPCR offers a streamlined and low-input alternative to conventional multi-step multiplex PCR methods. By combining reduced hands-on time and intrinsic contamination control with high analytical accuracy, this approach represents a practical refinement for targeted amplicon sequencing in diagnostic workflows.

## Introduction

While next-generation sequencing (NGS) has transitioned from fundamental research to routine clinical diagnostics [1–4], library preparation remains a formidable bottleneck. A primary challenge resides in the high failure rates associated with limited starting material. In a large-scale clinical study involving over 4,000 patients (the PROfound trial), nearly half of the tumor samples failed genomic testing, primarily due to inadequate DNA quantity or quality from tissue samples [5]. Second, the intrinsic complexity of conventional library preparation poses a significant barrier. Standard protocols typically involve a tedious series of enzymatic reactions including end-repair, A-tailing, and adapter ligation interspersed with multiple bead-based purification steps. Each transfer and purification step not only increases hands-on time and operational variability but also leads to cumulative sample loss, which is particularly detrimental for low-input specimens [6]. Furthermore, the potential contamination risk inherent to multi-step handling jeopardizes the accuracy of high-sensitivity assays such as minimal residual disease (MRD) detection [7]. Consequently, there is an urgent need for a streamlined, single-vessel strategy that integrates library construction into a simplified, one-step process to maximize sample recovery and ensure success even with minimal input DNA.

To address these challenges, current clinical workflows primarily employ liquid-phase hybrid capture and multiplex PCR-based enrichment. While hybrid capture is suitable for large-scale genomic regions, its adoption is hindered by a laborious multi-step process, high probe costs, and significant material loss during extensive purification cycles [6,8]. Multiplex PCR offers a more streamlined alternative with superior sensitivity for low-input samples. Notably, specialized techniques like Single-End Anchored Multiplex PCR (AMP) have been developed to detect gene fusions [9]. However, AMP and similar conventional multiplex methods still necessitate separate, sequential steps for adapter ligation and enrichment, leading to prolonged turnaround times and increased risks of cross-contamination inherent to multi-step processing [10–12]. Furthermore, existing simplified protocols often rely on integrated long primers [7], which are not only cost-prohibitive but also prone to synthesis-related biases that can compromise amplification efficiency in trace samples. Therefore, developing a simplified, cost-effective, and low-input-compatible closed-tube strategy that integrates these complex steps into a one-step process is essential for advancing NGS clinical applications [13,14].

To address these critical challenges, we developed a novel one-tube, one-step multiplex PCR (OS-MPCR) library enrichment technology. This approach utilizes a ligase-dependent and template-guided mechanism where a splint oligo serves as a scaffold to facilitate the precise coupling of adapters and target-specific primers. A notable feature of this method is its modular design, in which indexing adapters and primers are synthesized separately and then assembled during the reaction process. By integrating target enrichment and adapter ligation into a single thermal cycling session, the OS-MPCR method achieves a streamlined closed-tube operation. This advancement reduces hands-on time from 3 hours to just 20 minutes while effectively mitigating the risk of handling-associated contamination. Validation using standard reference materials demonstrated that the technology is compatible with inputs as low as 1 ng DNA and 5 ng RNA, showing the capability to detect SNV and Indel variants at frequencies as low as 1%. Furthermore, a comparative study using clinical FFPE samples showed that our OS-MPCR (employing DNA for SNV/Indel detection and RNA for fusion detection) achieved a 100% consistency rate with conventional liquid-phase hybrid capture. Consequently, this technology provides a contamination-resistant, cost-effective, and low-input-compatible solution that significantly streamlines the workflow for targeted amplicon sequencing in diagnostic applications.

## Materials and methods

### Reference materials and clinical specimens

To evaluate the analytical performance of the OS-MPCR assay, two types of reference materials were employed. Reference RNA samples (Seraseq^®^ FFPE Fusion RNA Reference Material Mix v4, SeraCare, Milford, MA, USA) were utilized, which contain known fusions and splicing variants. The absolute concentrations of the target transcripts in the undiluted positive reference were pre-determined by droplet digital PCR (ddPCR), yielding 132.7 copies/ng for *EML4-ALK*, 534.7 copies/ng for *CD74-ROS1*, and 407.7 copies/ng for *MET* exon 14 skipping. This positive reference RNA was then serially diluted with wild-type RNA to achieve specific mass fractions (5% and 1%, w/w) for sensitivity testing.

Reference DNA samples (GW-OGTM006, GeneWell, Shenzhen, China) were utilized as positive standards to analyze three distinct EGFR alterations: the L858R mutation, the T790M mutation, and the exon 19 deletion. This reference material was individually diluted with wild-type genomic DNA to achieve defined variant allele frequencies (VAFs) of 3%, 2%, and 1% for analytical validation.

A total of 114 retrospective clinical specimens were enrolled in this study to evaluate the feasibility of the detection method. The primary cohort consisted of 110 archived specimens from lung cancer patients, comprising 76 formalin-fixed paraffin-embedded (FFPE) DNA samples and 34 FFPE RNA samples. For additional exploratory validation, one pleural effusion sample and three blood samples were also collected and analyzed. To rigorously challenge the analytical limits and assess performance stability, the selection covered a broad dynamic range of target concentrations: it included both borderline cases near the limit of detection (LOD) and specimens with high signal abundance (60%−95%) secondary to gene copy number variations (CNVs).

This study was conducted in accordance with the Declaration of Helsinki and approved by the Medical Ethics Committee of Zibo First Hospital (Approval No. YXLL20240638). The research utilized existing, residual, and de-identified clinical specimens that had been previously archived for routine diagnostic purposes. Given the retrospective nature of the study and the fact that it posed no more than minimal risk to the subjects, a waiver of informed consent was formally granted by the Ethics Committee.

The research team accessed the archived samples and associated clinical data for research purposes from June 15, 2024, to December 31, 2025. All data were fully anonymized prior to the commencement of the study. At no point during or after data collection did the authors have access to any information that could identify individual participants, ensuring strict adherence to patient privacy and confidentiality protocols.

### Nucleic acid extraction and quantification

Genomic DNA from FFPE tissues was extracted using the HiPure FFPE DNA Kit (Magen, Guangzhou, China). For liquid biopsy specimens, circulating cell-free DNA (cfDNA) from plasma and pleural effusion was isolated using the HiPure Circulating DNA Kit (Magen). Total RNA was extracted from FFPE sections utilizing the RNApure FFPE Kit (Cwbio, Taizhou, China). All extraction procedures were performed following the manufacturers’ instructions. The concentration of the extracted nucleic acids was determined using a Qubit 4.0 Fluorometer with the dsDNA HS or RNA HS Assay Kits (Thermo Fisher Scientific, Singapore).

### Design of OS-MPCR panels and primers

Two targeted panels were developed: a DNA panel for SNV/Indel detection and an RNA panel for fusion and exon skipping identification. The full-length primer construction utilizes a modular assembly strategy comprising three components: specific primers, indexing adapters, and splint oligos.

Each specific primer contains a sequence for genomic recognition and a fixed segment serving as a linkage site. The forward specific primers are 5’-phosphorylated (5’-P-) to enable ligation. The indexing adapters, carrying i5 or i7 barcodes, also possess a corresponding linkage site. The splint oligo acts as a bridge, with its two halves being complementary to the linkage sites of the specific primer and the indexing adapter, respectively. During the reaction, the splint oligo brings the specific primer and indexing adapter together, allowing them to be covalently joined by a ligase. This assembly forms a full-length functional primer that initiates the subsequent multiplex PCR amplification. Detailed sequences are provided in S1-S3 Tables.

### Library preparation via OS-MPCR technology

For RNA samples, the first-strand cDNA was synthesized using the Hieff NGS® 1st cDNA Synthesis Kit (Yeasen, Shanghai, China). For both DNA and cDNA templates, the OS-MPCR reaction was conducted for each individual sample in an independent single-tube format, enabling seamless integration of modular primer assembly and target amplification. The detailed reaction components and thermal cycling profiles are summarized in Table 1 and Table 2, respectively.

**Table 1:** Optimized reaction components for the OS-MPCR assay.

Components	Volume (Total volume: 25 μL)
Input DNA/cDNA	10 ng/15 μL
5 × Assemplex Multiplex Buffer	5 μL
Assemplex Multiplex Enzyme	1 μL
Specific Primer Pool (DNA panel: 4 μM; RNA Panel: 5 μM)	DNA panel: 2.6 μL; RNA Panel: 1.5 μL
Index Primer (DNA panel: 4 μM; RNA Panel: 5 μM)	DNA panel: 2.6 μL; RNA Panel: 1.5 μL
Nuclease-free water	To 25 μL

**Table 2:** Thermal cycling conditions for the OS-MPCR library preparation.

Temperature	Time	Cycle number
37℃	10min	1
95℃	3min	1
95℃	30sec	40
60℃	90sec	
72℃	1min	
72℃	2min	1
4℃	hold	1

The reaction program for each individual library comprised two consecutive stages: an initial ligation phase to facilitate the in situ assembly of full-length functional primers, followed by a multiplex PCR phase for targeted enrichment. The resulting library from each sample was purified using 0.8 × VAHTS DNA Clean Beads (Vazyme, Nanjing, China) and eluted in 20 μL of nuclease-free water. Library quantification and size distribution analysis were performed individually for each purified library using a Qubit 4.0 Fluorometer (Thermo Fisher Scientific, Singapore) and a Qsep1 Fragment Analyzer (BiOptic Inc., Changzhou, China), respectively, ensuring stringent quality control and normalization before pooling for downstream sequencing.

### Library preparation via liquid-phase hybrid capture

To evaluate the performance of the OS-MPCR assay, parallel library preparation was performed using liquid-phase hybrid capture technology at Shanghai Yijian Medical Laboratory Co., Ltd., China.

#### Pre-library construction.

For FFPE tissue DNA, pre-libraries were constructed using the DNA Fragmentation & Library Prep Kit (YJMed, Shanghai, China), which integrates enzymatic fragmentation, end-repair, and adapter ligation. For circulating DNA from blood and pleural effusion samples, pre-libraries were prepared utilizing the VAHTS Universal DNA Library Prep Kit for Illumina^®^ V3 (Vazyme, Nanjing, China).

#### Target enrichment and quality control.

The enrichment of target regions was conducted using the Target Capture Hybridization & Wash Kit (YJMed, Shanghai, China) following the manufacturer’s instructions. Briefly, the pre-libraries were hybridized with biotinylated probes, followed by streptavidin-coated magnetic bead capture and subsequent wash steps to remove off-target fragments. The final captured libraries were amplified and then quantified using a Qubit 4.0 Fluorometer (Thermo Fisher Scientific, Singapore). The size distribution of the libraries was assessed via a Qsep400 Fragment Analyzer (BiOptic Inc., Changzhou, China).

### Bioinformatics processing

Pooled libraries were sequenced on DNBSEQ-T7 (MGI Tech Co., Ltd, Shenzhen, China) by paired-end, with approximately 0.01−2 Gb of sequencing data generated per sample. Adapter sequences and poly-G sequences at both ends were removed before alignment by CutAdapt (version 4.9) [15]. Read pairs with insert size < 50 bp were discarded. Reads were aligned by using BWA-mem (version 0.7.18) [16]. To identify fusion and MET 14 exon skipping, FASTQ files were aligned to the targeted fused transcript and MET transcript (with or without MET exon 14 skipping). To identify SNVs and Indels, FASTQ files were aligned to the hg19 version of the human genome. Vardict (version 1.5.8) [17] was used for SNV calling and Indel events detecting. SNV and Indels were annotated with ANNOVAR (version 2016-02-01) [18] and SnpEff (version 4.3t) [19]. Only variant annotations relative to the canonical transcript for each gene were reported. SNV and Indels were filtered by a custom script to remove common population polymorphisms and background noise mutations. All mutations were manually reviewed by using the Integrated Genomics Viewer [20].

### Analytical performance evaluation using reference materials

To rigorously evaluate the analytical performance of the OS-MPCR assay, serial dilution studies were conducted using standardized DNA and RNA reference materials.

#### Performance validation of the DNA panel.

The impact of DNA input quantity and variant frequency on assay sensitivity was systematically analyzed. To determine the minimum required DNA input, reference materials (GW-OGTM006, GeneWell, Shenzhen, China) were diluted with wild-type DNA to achieve VAFs of 1%, 2%, and 3%. For each VAF level, a range of DNA inputs (1 ng, 5 ng, 10 ng, and 20 ng) was tested.

#### Performance validation of the RNA panel.

For the RNA panel, the analytical sensitivity was assessed using the Seraseq® FFPE Fusion RNA Reference Material Mix v4. The positive reference RNA was diluted with wild-type RNA to mass fractions of 1% and 5%. To evaluate the assay’s robustness across varying template concentrations, these dilutions were tested with total RNA inputs of 5 ng and 10 ng, respectively. These experiments were designed to establish the lower bound of detection for fusion transcripts and exon skipping events.

### Concordance analysis with liquid-phase hybrid capture technology

To validate the clinical utility of the OS-MPCR assay, a head-to-head concordance analysis was performed using specimens previously characterized by an independent clinical laboratory using liquid-phase hybrid capture. The DNA panel’s performance was evaluated using 76 genomic DNA samples, while the RNA panel’s accuracy was assessed using 34 clinical RNA samples. These specimens represented a diverse range of oncogenic alterations, including SNVs, Indels, fusions, and exon skipping events. The results obtained from the OS-MPCR assay were compared with the medical laboratory findings to determine the concordance between the two methodologies.

### Feasibility exploration in alternative sample types

To assess the clinical versatility of the DNA panel, feasibility testing was conducted using pleural effusion and blood-derived specimens.

First, a pleural effusion sample, previously confirmed by the medical laboratory to harbor the EGFR T790M mutation, was utilized for a sensitivity challenge. The mutant DNA was serially diluted with wild-type genomic DNA to generate a gradient of VAFs at 1%, 0.5%, and 0.1%. These diluted samples were then processed through the DNA panel workflow to evaluate the detection limit in a complex matrix.

Second, the panel’s performance in liquid biopsy was further validated using three clinical blood samples with known low-abundance variants, as pre-verified by the medical laboratory. These experiments were designed to confirm the robust performance of the OS-MPCR technology in identifying trace somatic mutations across different clinical specimen formats.

### Statistical analysis

Data are presented as the mean ± standard error of the mean (SEM). All experiments were performed in triplicate to ensure reproducibility. Graphical representations, including box-plots and scatter plots, were generated using GraphPad Prism version 10 (GraphPad Software, San Diego, CA, USA).

To evaluate the diagnostic performance of the OS-MPCR assay, key validation metrics were defined and calculated as follows: Accuracy = (TP + TN) / (TP + FP + TN + FN); Sensitivity = TP / (TP + FN); Specificity = TN / (TN + FP). In these formulas, TP, TN, FP, and FN represent true positives, true negatives, false positives, and false negatives, respectively.

## Results

### Design of the OS-MPCR technology

**Fig 1:**
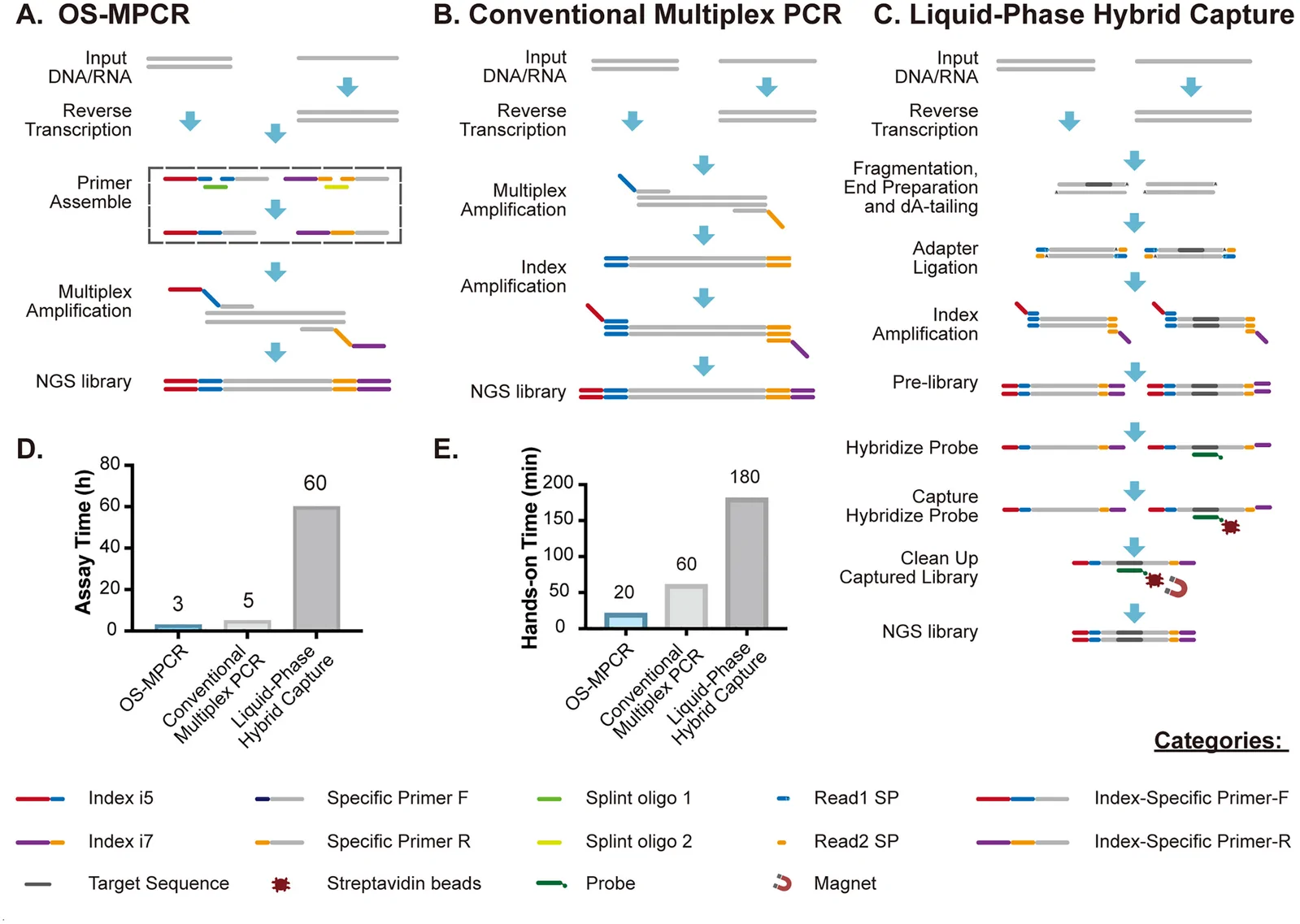
Procedural schematics and efficiency comparisons of library preparation methods. Comparison between One-Step Multiplex PCR (OS-MPCR) (A), conventional multiplex PCR (B), and liquid-phase hybrid capture (C). The OS-MPCR workflow features a unique Primer Assemble phase facilitated by splint oligos to bridge index adapters and specific primers in a single reaction. D-E. Efficiency metrics: Quantitative comparison of the total assay turnaround time (D) and manual hands-on time (E) among the three methods. OS-MPCR significantly reduces the library preparation, requiring only 20 minutes of manual intervention.

OS-MPCR was designed to integrate target enrichment and sequencing adapter addition into a streamlined, single-reaction format. The fundamental mechanism relies on a splint-oligo mediated ligation strategy that assembles functional long primers (Fig 1A). As illustrated in the schematic, the reaction system comprises adapter primers (Index i5 and i7), gene-specific primers (Primer-F and Primer-R), and specialized splint oligos. During the Primer Assemble phase, the splint oligos act as molecular scaffolds, precisely aligning the adapter primers with the specific primers. This alignment facilitates their ligation into complete Index-Specific Primers (F and R), which then drive the subsequent multiplex amplification. By coupling ligation and amplification in one step, OS-MPCR bypasses the need for the multi-stage library construction typically required in NGS workflows.

### The OS-MPCR technology reduces time consumption

To evaluate the practical operational advantages of the OS-MPCR system, we performed a quantitative workflow comparison against conventional multiplex PCR, while referencing liquid-phase hybrid capture as the industry gold standard for comprehensive genomic profiling (Fig 1D, E). Within the target-specific amplicon framework, the total assay time for OS-MPCR is optimized to just 3 hours, achieving a substantial reduction compared to the 5 hours required for conventional multi-step multiplex PCR, which typically involves separate amplification and index ligation stages (Fig 1D). Correspondingly, the hands-on time for OS-MPCR was streamlined to only 20 minutes, representing a threefold efficiency gain over the 60 minutes required for conventional multiplex PCR (Fig 1E). This dramatic reduction in labor and time is achieved by eliminating the multi-step transfers and purification cycles inherent to traditional workflows into a single-tube, fully integrated reaction. For context, while liquid-phase hybrid capture remains the standard for broad, unbiased sequencing, its intrinsic reliance on overnight hybridization and complex multi-step “Capture & Wash” protocols demands significantly longer total assay times (60 hours) and intensive manual labor (180 minutes) (Fig 1D, E).

### Performance analysis of the OS-MPCR technology using standard reference

**Fig 2:**
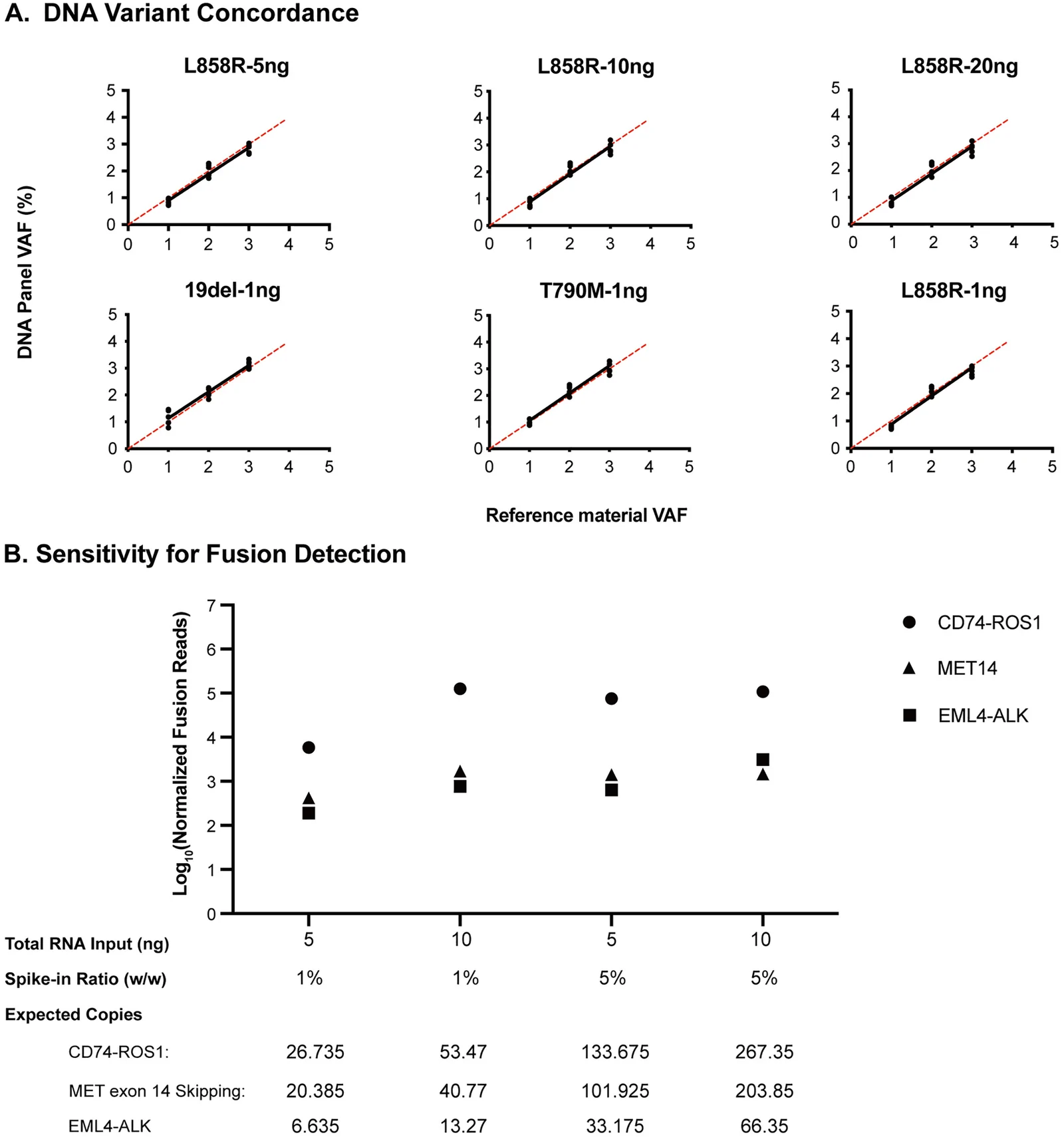
Analytical Performance and Sensitivity Analysis Using Reference Materials. A. DNA Variant Concordance: Linear correlation analysis between expected and observed Variant Allele Frequencies (VAF) for clinical hotspots (EGFR L858R, 19del, T790M) across varying DNA inputs (1-20 ng). The OS-MPCR system demonstrates high quantitative accuracy at VAF levels as low as 1%. B. Sensitivity for Fusion Detection: Detection of RNA fusion events (*CD74-ROS1*, *MET14* skipping, *EML4-ALK*) at 1% and 5% spike-in ratios (w/w). The Log_10_ transformed normalized fusion reads reflect robust detection sensitivity across different total RNA inputs (5-10 ng), with performance at low inputs (5 ng, 1%) tightly coupled to the absolute expected copy numbers of the target molecules.

To evaluate the analytical accuracy and sensitivity of the OS-MPCR system, we tested the platform using standard reference materials across various DNA and RNA input levels (Fig 2, S1 Fig). For DNA variant detection, we assessed the concordance of variant allele frequencies (VAF) for clinical markers including EGFR L858R, Exon 19 deletion (19del), and T790M. As shown in Fig 2A, the VAF values measured by OS-MPCR demonstrated exceptional linearity with the expected reference frequencies across a range of 1% to 3% VAF, even with an input as low as 1 ng of DNA. This high degree of correlation confirms the quantitative reliability of OS-MPCR in detecting low-frequency mutations. Furthermore, we characterized the system’s sensitivity for fusion detection using RNA reference materials for *CD74-ROS1*, *MET14* skipping, and *EML4-ALK* (Fig 2B). The platform successfully identified all fusion events at 1% and 5% spike-in ratios (w/w) with total RNA inputs ranging from 5 ng to 10 ng. Notably, after normalizing for sequencing depth, the Log_10_ transformed fusion-supporting reads for the 5% ratio remained highly stable and comparable between the 5 ng and 10 ng inputs. At the lower 1% ratio, a slight decrease in normalized reads was observed at 5 ng compared to 10 ng input, reflecting the stochastic sampling effects and reduced molecular conversion efficiency inherent to ultra-low absolute copy numbers (e.g., ~ 6.6 copies for *EML4-ALK*). These results underscore the robust sensitivity, technical transparency, and diagnostic potential of OS-MPCR for high-precision genomic profiling.

### QC performance of OS-MPCR libraries based on clinical samples

**Fig 3:**
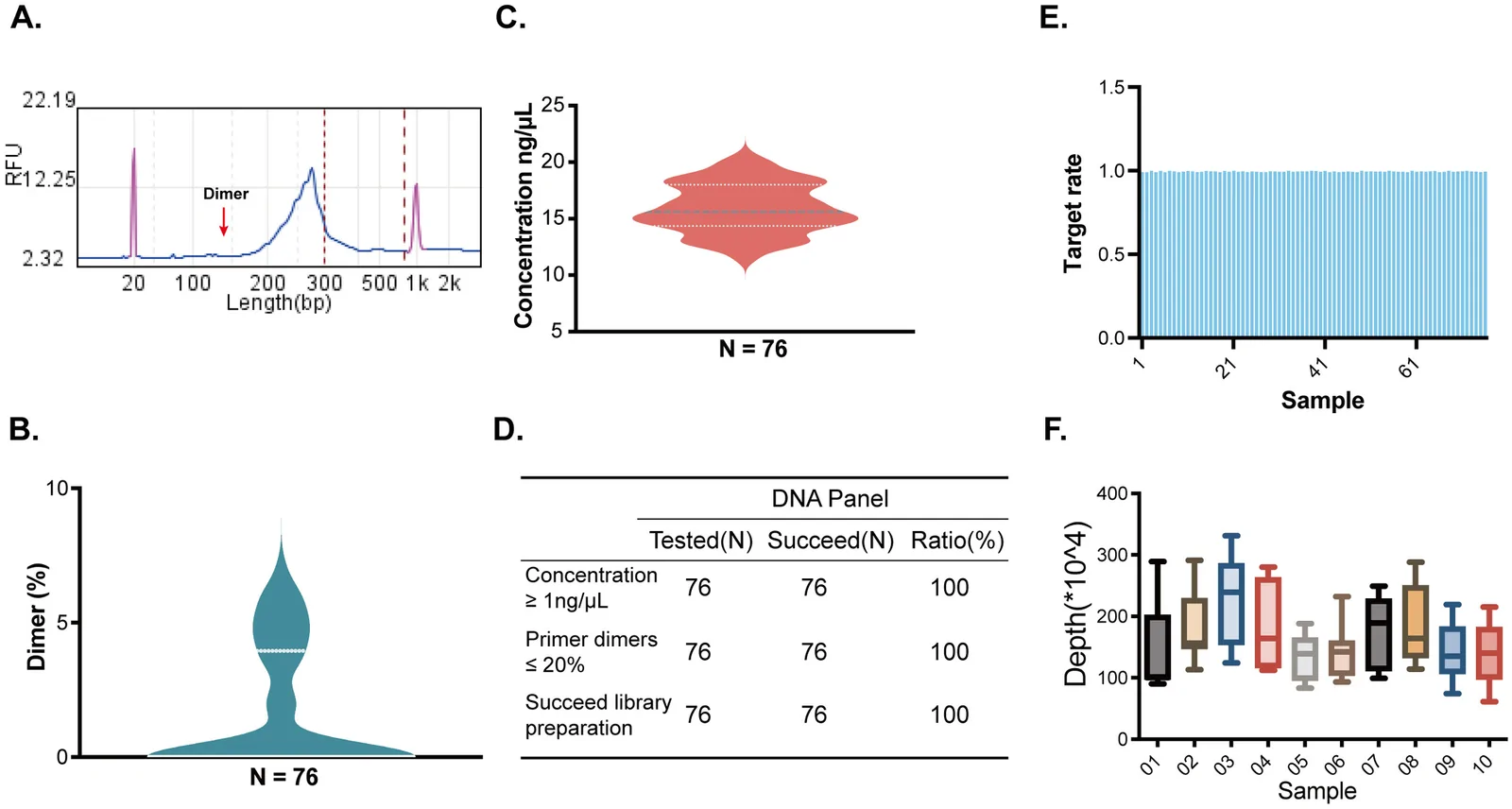
Quality Control (QC) and Sequencing Performance of OS-MPCR in Clinical DNA Samples. A-B. Fragment Analysis: Representative electropherogram showing a characteristic broad library peak centered at approximately 290 bp, which is typical for multiplex PCR amplicons of varying lengths (A). The distribution of primer dimer ratios was consistently maintained below 8% across 76 clinical samples (B). C. Distribution of Library Concentrations: Violin plot illustrating the library yield for the 76 DNA specimens (N = 76) prepared by OS-MPCR. The tight clustering of data points demonstrates the high consistency and reproducibility of the workflow, with all yields being sufficient for subsequent sequencing. D. Library Success Rate: Summary table indicating a 100% success rate based on library concentration, primer dimer control, and overall library preparation across all 76 tested samples. E. Target Rate Stability: Profiling of sequencing specificity across the clinical cohort. The OS-MPCR system consistently maintained a near 1.0 (100%) target rate across all 76 samples, demonstrating exceptional enrichment specificity. F. Sequencing Depth: Box plot showing the uniform distribution of mean sequencing depth across clinical samples, with all samples exceeding 100 × 10^4^.

The robust performance of the OS-MPCR system was further validated through a comprehensive quality control (QC) analysis of 76 clinical DNA samples (N = 76). Representative electropherograms and fragment analysis revealed that OS-MPCR libraries exhibited a characteristic broad peak centered at approximately 290 bp, which is typical for multiplex PCR amplicons of varying lengths. The primer dimer rate was strictly maintained below 8% across the entire cohort, demonstrating the exceptional efficiency and specificity of the one-step integrated reaction (Fig 3A, 3B). The concentration of the resulting libraries showed a highly consistent distribution, with all yields being sufficient for subsequent high-depth sequencing and contributing to a 100% library preparation success rate based on our stringent QC thresholds (Fig 3C, 3D). Furthermore, evaluation of sequencing specificity demonstrated that the target rate for OS-MPCR remained remarkably stable and near 1.0 (100%) across all clinical samples (Fig 3E). The sequencing depth also demonstrated high uniformity across samples, with the mean depth consistently exceeding 100 × 10^4^, thereby ensuring reliable and high-precision variant calling across the entire panel (Fig 3F).

### Concordance between OS-MPCR and hybrid capture in clinical samples

**Fig 4:**
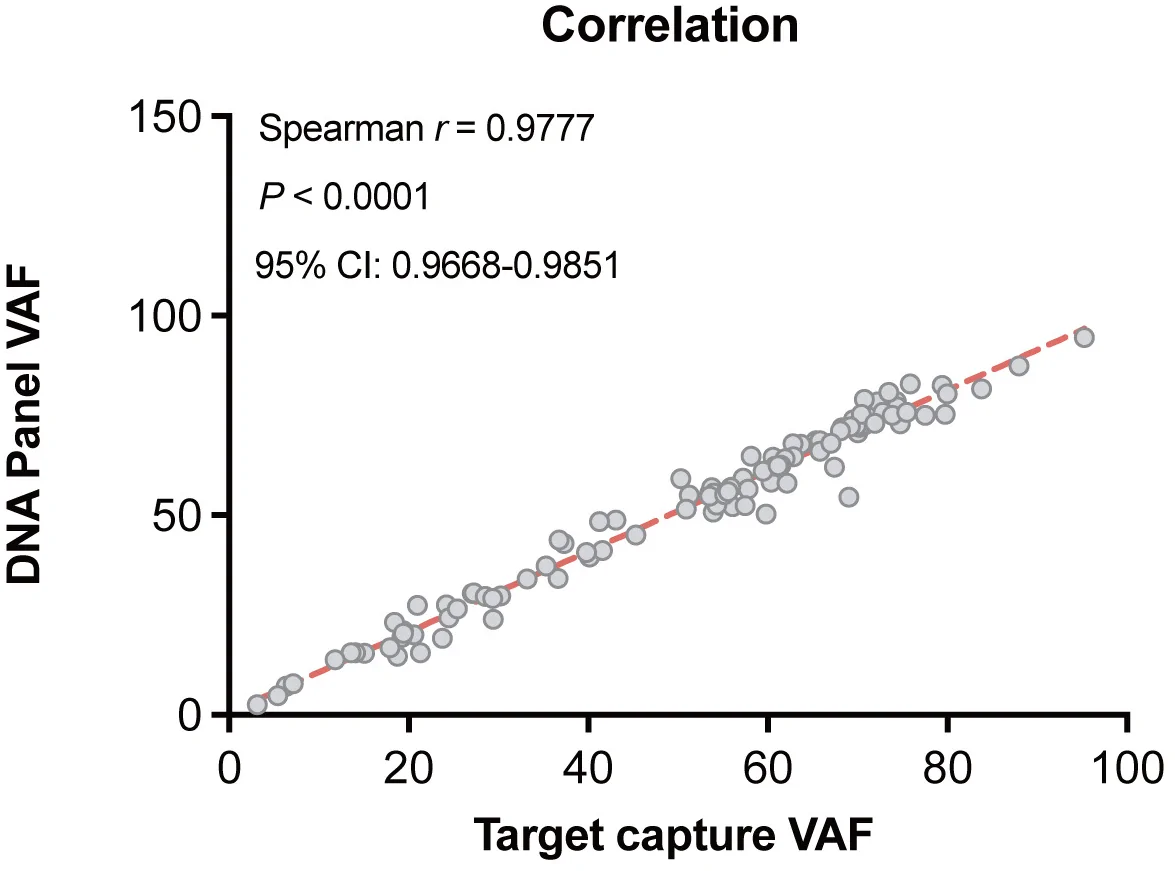
Correlation analysis of mutation abundances between the DNA panel using the OS-MPCR and the Liquid-Phase Hybrid Capture technology.

The clinical reliability of the OS-MPCR technology was evaluated through a head-to-head concordance analysis with the well-established liquid-phase hybrid capture method using clinical specimens. For DNA variant calling, OS-MPCR demonstrated exceptional quantitative consistency with the hybrid capture platform, as evidenced by a strong positive correlation in VAF values across all detected mutations (Spearman *r* = 0.9777, 95% CI: 0.9668-0.9851, *P* < 0.0001) (Table 3, Fig 4). The diagnostic performance for pathogenic hotspots achieved 100% sensitivity and specificity across 76 tested samples, with no false positives or false negatives recorded. This high degree of concordance confirms that OS-MPCR provides diagnostic depth and accuracy comparable to well-established capture-based methods while significantly reducing technical complexity.

**Table 3:** Concordance of SNV/Indel analysis of DNA panel using OS-MPCR compared with liquid-phase hybrid capture technology.

Gene	pathogenic hotspot	TP	FP	TN	FN	Total	PPV	NPV	Accuracy	Sensitivity	Specificity
EGFR	p.L858R	8	0	68	0	76	100% (8/8, 95% CI: 67.559%−100%)	100% (68/68, 95% CI: 94.653%−100%)	100% (76/76, 95% CI: 95.189%−100%)	100% (8/8, 95% CI: 67.559%−100%)	100% (68/68, 95% CI: 94.653%−100%)
EGFR	19 del	7	0	69	0	76	100% (7/7, 95% CI: 64.567%−100%)	100% (69/69, 95% CI: 94.726%−100%)	100% (76/76, 95% CI: 95.189%−100%)	100% (7/7, 95% CI: 64.567%−100%)	100% (69/69, 95% CI: 94.726%−100%)
EGFR	p.T790M	4	0	72	0	76	100% (4/4, 95% CI: 51.011%−100%)	100% (72/72, 95% CI: 94.935%−100%)	100% (76/76, 95% CI: 95.189%−100%)	100% (4/4, 95% CI: 51.011%−100%)	100% (72/72, 95% CI: 94.935%−100%)
EGFR	p.C797S	3	0	73	0	76	100% (3/3, 95% CI: 43.850%−100%)	100% (73/73, 95% CI: 95.001%−100%)	100% (76/76, 95% CI: 95.189%−100%)	100% (3/3, 95% CI: 43.850%−100%)	100% (73/73, 95% CI: 95.001%−100%)
EGFR	p.G719A	3	0	73	0	76	100% (3/3, 95% CI: 43.850%−100%)	100% (73/73, 95% CI: 95.001%−100%)	100% (76/76, 95% CI: 95.189%−100%)	100% (3/3, 95% CI: 43.850%−100%)	100% (73/73, 95% CI: 95.001%−100%)
EGFR	p.G719S	3	0	73	0	76	100% (3/3, 95% CI: 43.850%−100%)	100% (73/73, 95% CI: 95.001%−100%)	100% (76/76, 95% CI: 95.189%−100%)	100% (3/3, 95% CI: 43.850%−100%)	100% (73/73, 95% CI: 95.001%−100%)
EGFR	p.E709A	3	0	73	0	76	100% (3/3, 95% CI: 43.850%−100%)	100% (73/73, 95% CI: 95.001%−100%)	100% (76/76, 95% CI: 95.189%−100%)	100% (3/3, 95% CI: 43.850%−100%)	100% (73/73, 95% CI: 95.001%−100%)
EGFR	p.R776C	3	0	73	0	76	100% (3/3, 95% CI: 43.850%−100%)	100% (73/73, 95% CI: 95.001%−100%)	100% (76/76, 95% CI: 95.189%−100%)	100% (3/3, 95% CI: 43.850%−100%)	100% (73/73, 95% CI: 95.001%−100%)
EGFR	p.L861Q	5	0	71	0	76	100%(5/5, 95% CI: 56.552%−100%)	100%(71/71, 95% CI: 94.867%−100%)	100% (76/76, 95% CI: 95.189%−100%)	100%(5/5, 95% CI: 56.552%−100%)	100%(71/71, 95% CI: 94.867%−100%)
EGFR	p.H835L	5	0	71	0	76	100%(5/5, 95% CI: 56.552%−100%)	100%(71/71, 95% CI: 94.867%−100%)	100% (76/76, 95% CI: 95.189%−100%)	100%(5/5, 95% CI: 56.552%−100%)	100%(71/71, 95% CI: 94.867%−100%)
BRAF	p.V600E	6	0	70	0	76	100%(6/6, 95% CI: 60.967%−100%)	100%(70/70, 95% CI: 94.798%−100%)	100% (76/76, 95% CI: 95.189%−100%)	100%(6/6, 95% CI: 60.967%−100%)	100%(70/70, 95% CI: 94.798%−100%)
BRAF	p.K601E	3	0	73	0	76	100% (3/3, 95% CI: 43.850%−100%)	100% (73/73, 95% CI: 95.001%−100%)	100% (76/76, 95% CI: 95.189%−100%)	100% (3/3, 95% CI: 43.850%−100%)	100% (73/73, 95% CI: 95.001%−100%)
BRAF	p.D594N	3	0	73	0	76	100% (3/3, 95% CI: 43.850%−100%)	100% (73/73, 95% CI: 95.001%−100%)	100% (76/76, 95% CI: 95.189%−100%)	100% (3/3, 95% CI: 43.850%−100%)	100% (73/73, 95% CI: 95.001%−100%)
ERBB2	20 ins	8	0	68	0	76	100% (8/8, 95% CI: 67.559%−100%)	100% (68/68, 95% CI: 94.653%−100%)	100% (76/76, 95% CI: 95.189%−100%)	100% (8/8, 95% CI: 67.559%−100%)	100% (68/68, 95% CI: 94.653%−100%)
ERBB2	p.V777L	3	0	73	0	76	100% (3/3, 95% CI: 43.850%−100%)	100% (73/73, 95% CI: 95.001%−100%)	100% (76/76, 95% CI: 95.189%−100%)	100% (3/3, 95% CI: 43.850%−100%)	100% (73/73, 95% CI: 95.001%−100%)
KRAS	p.G12A	3	0	73	0	76	100% (3/3, 95% CI: 43.850%−100%)	100% (73/73, 95% CI: 95.001%−100%)	100% (76/76, 95% CI: 95.189%−100%)	100% (3/3, 95% CI: 43.850%−100%)	100% (73/73, 95% CI: 95.001%−100%)
KRAS	p.G12C	3	0	73	0	76	100% (3/3, 95% CI: 43.850%−100%)	100% (73/73, 95% CI: 95.001%−100%)	100% (76/76, 95% CI: 95.189%−100%)	100% (3/3, 95% CI: 43.850%−100%)	100% (73/73, 95% CI: 95.001%−100%)
KRAS	p.G12D	3	0	73	0	76	100% (3/3, 95% CI: 43.850%−100%)	100% (73/73, 95% CI: 95.001%−100%)	100% (76/76, 95% CI: 95.189%−100%)	100% (3/3, 95% CI: 43.850%−100%)	100% (73/73, 95% CI: 95.001%−100%)
KRAS	p.G12V	3	0	73	0	76	100% (3/3, 95% CI: 43.850%−100%)	100% (73/73, 95% CI: 95.001%−100%)	100% (76/76, 95% CI: 95.189%−100%)	100% (3/3, 95% CI: 43.850%−100%)	100% (73/73, 95% CI: 95.001%−100%)
KRAS	p.G13D	3	0	73	0	76	100% (3/3, 95% CI: 43.850%−100%)	100% (73/73, 95% CI: 95.001%−100%)	100% (76/76, 95% CI: 95.189%−100%)	100% (3/3, 95% CI: 43.850%−100%)	100% (73/73, 95% CI: 95.001%−100%)
NRAS	p.G12D	3	0	73	0	76	100% (3/3, 95% CI: 43.850%−100%)	100% (73/73, 95% CI: 95.001%−100%)	100% (76/76, 95% CI: 95.189%−100%)	100% (3/3, 95% CI: 43.850%−100%)	100% (73/73, 95% CI: 95.001%−100%)
NRAS	p.G13D	3	0	73	0	76	100% (3/3, 95% CI: 43.850%−100%)	100% (73/73, 95% CI: 95.001%−100%)	100% (76/76, 95% CI: 95.189%−100%)	100% (3/3, 95% CI: 43.850%−100%)	100% (73/73, 95% CI: 95.001%−100%)
PIK3CA	p.E542K	5	0	71	0	76	100%(5/5, 95% CI: 56.552%−100%)	100%(71/71, 95% CI: 94.867%−100%)	100% (76/76, 95% CI: 95.189%−100%)	100%(5/5, 95% CI: 56.552%−100%)	100%(71/71, 95% CI: 94.867%−100%)
PIK3CA	p.E545K	6	0	70	0	76	100%(6/6, 95% CI: 60.967%−100%)	100%(70/70, 95% CI: 94.798%−100%)	100% (76/76, 95% CI: 95.189%−100%)	100%(6/6, 95% CI: 60.967%−100%)	100%(70/70, 95% CI: 94.798%−100%)
PIK3CA	p.P539R	3	0	73	0	76	100% (3/3, 95% CI: 43.850%−100%)	100% (73/73, 95% CI: 95.001%−100%)	100% (76/76, 95% CI: 95.189%−100%)	100% (3/3, 95% CI: 43.850%−100%)	100% (73/73, 95% CI: 95.001%−100%)

TP = true positive, TN = true negative, FP = false positive, FN = false negative, PPV = positive predictive value, NPV = negative predictive value. 95% confidence intervals (CIs) for diagnostic metrics were calculated using the Wilson score method.

Regarding RNA fusion detection, OS-MPCR exhibited robust diagnostic performance across a variety of clinically relevant fusion events. In a cohort of 34 samples, the OS-MPCR system achieved 100% accuracy, sensitivity, and specificity for all target fusion genes (Table 4). These results underscore the technical maturity of the OS-MPCR platform in identifying complex structural rearrangements and its readiness for high-precision clinical molecular diagnostics.

**Table 4:** Concordance of fusion analysis of RNA panel using the OS-MPCR compared with liquid-phase hybrid capture technology.

Fusion Gene	TP	FP	TN	FN	Total	PPV	NPV	Accuracy	Sensitivity	Specificity
EML4(Exon2)-ALK(Exon20)	5	0	29	0	34	100% (5/5, 95% CI: 56.552%−100%)	100% (29/29, 95% CI: 88.303%−100%)	100% (34/34, 95% CI: 89.849%−100%)	100% (5/5, 95% CI: 56.552%−100%)	100% (29/29, 95% CI: 88.303%−100%)
EML4(Exon20)-ALK(Exon20)	5	0	29	0	34	100% (5/5, 95% CI: 56.552%−100%)	100% (29/29, 95% CI: 88.303%−100%)	100% (34/34, 95% CI: 89.849%−100%)	100% (5/5, 95% CI: 56.552%−100%)	100% (29/29, 95% CI: 88.303%−100%)
EML4(Exon13)-ALK(Exon20)	5	0	29	0	34	100% (5/5, 95% CI: 56.552%−100%)	100% (29/29, 95% CI: 88.303%−100%)	100% (34/34, 95% CI: 89.849%−100%)	100% (5/5, 95% CI: 56.552%−100%)	100% (29/29, 95% CI: 88.303%−100%)
EML4(Exon6)-ALK(Exon20)	5	0	29	0	34	100% (5/5, 95% CI: 56.552%−100%)	100% (29/29, 95% CI: 88.303%−100%)	100% (34/34, 95% CI: 89.849%−100%)	100% (5/5, 95% CI: 56.552%−100%)	100% (29/29, 95% CI: 88.303%−100%)
KIF5B(Exon15)-RET(Exon12)	4	0	30	0	34	100% (4/4, 95% CI: 51.011%−100%)	100% (30/30, 95% CI: 88.649%−100%)	100% (34/34, 95% CI: 89.849%−100%)	100% (4/4, 95% CI: 51.011%−100%)	100% (30/30, 95% CI: 88.649%−100%)
*MET14* skipping	6	0	28	0	34	100% (6/6, 95% CI: 60.967%−100%)	100% (28/28, 95% CI: 87.936%−100%)	100% (34/34, 95% CI: 89.849%−100%)	100% (6/6, 95% CI: 60.967%−100%)	100% (28/28, 95% CI: 87.936%−100%)
CD74(Exon6)-ROS1(Exon34)	4	0	30	0	34	100% (4/4, 95% CI: 51.011%−100%)	100% (30/30, 95% CI: 88.649%−100%)	100% (34/34, 95% CI: 89.849%−100%)	100% (4/4, 95% CI: 51.011%−100%)	100% (30/30, 95% CI: 88.649%−100%)

TP = true positive, TN = true negative, FP = false positive, FN = false negative, PPV = positive predictive value, NPV = negative predictive value. 95% confidence intervals (CIs) for diagnostic metrics were calculated using the Wilson score method.

## Discussion

Targeted NGS has become a cornerstone of precision oncology, yet workflow optimization remains a critical focus. The concept framework of utilizing splint oligonucleotides to facilitate adapter ligation has been successfully implemented in platforms such as SPLAT for bisulfite sequencing [21] and SRSLY for single-stranded DNA library preparation [22]. While these pioneering methods demonstrated the utility of splint-mediated ligation in specialized genomic contexts, their application has historically been distinct from high-throughput targeted clinical diagnostics. Currently, the field remains divided between the breadth of hybrid capture and the speed of conventional amplicon-based enrichment [23]. While comprehensive panels like TruSight Oncology 500 offer extensive genomic profiling [24–26], they often require relatively high nucleic acid inputs (≥10−30 ng) and involve multi-step, labor-intensive library preparation. In this study, we demonstrate that our One-Step Multiplex PCR (OS-MPCR) strategy provides a practical and efficient alternative within its targeted scope. By uniquely integrating splint-mediated assembly with multiplex amplification into a single, closed-tube reaction, OS-MPCR offers a streamlined, low-input workflow optimized for targeted oncology panels without compromising analytical sensitivity or clinical concordance.

Furthermore, exploratory testing with expanded random primer designs under high-multiplex conditions demonstrated robust performance, consistently maintaining low primer-dimer ratios and high target rates (S1 File). To contextualize its utility, we have summarized the distinct performance profiles and optimal clinical applications of major NGS library preparation technologies (Table 5).

**Table 5:** Core performance profiles and optimal application scenarios of major NGS library preparation technologies.

Technology	Turnaround Time	Target Pan-Throughput Scalability	Structural Variant (SV) Discovery Capability	Operational Complexity	Optimal Application Scenario
OS-MPCR (Our Method)	Rapid (~3 hours)	Low-to-Medium (Up to hundreds of targets)	Predefined/Known Fusions Only	Minimal (Single-tube, integrated)	Urgent oncology profiling (e.g., companion diagnostics for critical hotspots); Rapid screening of precious clinical specimens with ultra-low nucleic acid input.
Conventional Multiplex PCR	Fast (~5 hours)	Low-to-Medium	Predefined/Known Fusions Only	Medium (Requires multi-step purification)	Routine target-specific profiling; Low-to-medium-throughput hotspot mutation genotyping; Standard diagnostic labs with conventional PCR infrastructure.
Liquid-Phase Hybrid Capture	Slow (~60 hours)	High-to-Ultra-high (Exomes to Genomes)	Strong (Capable of novel fusion/breakpoint discovery)	High (Intensive “Capture & Wash” steps)	Comprehensive genomic profiling (CGP); Large-scale biomarker assessment (e.g., TMB, MSI); Novel structural variant/fusion discovery and exploratory research.

### Efficiency and contamination control

A primary bottleneck in clinical NGS is the labor-intensive nature of conventional library preparation workflows. Compared with traditional liquid-phase hybrid capture and even standard multiplex PCR, which require extended turnaround times and multi-step manipulations, OS-MPCR significantly shortens the workflow. As shown in Fig 1D and 1E, OS-MPCR compresses the total assay time to approximately 3 hours and requires only 20 minutes of manual hands-on time, representing a substantial improvement over the 60-minute hands-on time typical of conventional multiplex PCR. Beyond operational efficiency, the molecular design of OS-MPCR integrates adapter ligation and target amplification into a single thermal cycling session. By eliminating intermediate purification and transfer steps required by standard multiplex methods, OS-MPCR establishes a simplified closed-tube reaction environment. This single-vessel strategy effectively mitigates the risk of handling-associated cross-contamination, addressing a persistent challenge in high-throughput clinical diagnostic laboratories.

### Robust performance with ultra-low input

Tissue availability often limits NGS applications [27–29], particularly when pathology workflows exhaust samples during initial diagnosis. Our findings demonstrate that OS-MPCR achieves actionable sensitivity at inputs significantly lower than current industry standards. While commercial hybrid capture typically requires ~40 ng DNA/20 ng RNA [24], and established methods like AMP-PCR or existing “one-step” patents necessitate 10−25 ng DNA and 20−30 ng RNA [9,30], OS-MPCR reliably identifies 1% VAF SNVs/Indels with only 1 ng DNA and detects critical fusions with 5 ng RNA (Fig 2). This 5-to-10-fold reduction in input requirement is achieved without compromising library quality. Clinical cohort analysis (N = 76) demonstrated highly consistent library yields and a stable, near 1.0 (100%) target rate, confirming the robust efficiency and high specificity of the platform under low-input clinical conditions (Fig 3).

### Clinical reliability and future directions

The 100% concordance observed between OS-MPCR and well-established liquid-phase hybrid capture methods across clinical FFPE cohorts underscores its readiness for routine diagnostics. The strong correlation in variant abundance (Spearman *r* = 0.9777, 95% CI: 0.9668-0.9851, *P* < 0.0001) confirms that the simplified workflow does not sacrifice quantitative accuracy (Fig 4). Moreover, the successful detection of low-abundance variants (0.1%−1%) in pleural effusion and blood samples (S4-S5 Tables) suggests potential applications in liquid biopsy and future minimal residual disease (MRD) monitoring [31]. We hypothesize that this exceptional sensitivity at low allele frequencies stems from the minimal template input requirements of the OS-MPCR chemistry, which minimizes sample loss and maximizes the conversion efficiency of rare target molecules. However, some limitations remain. First, because all molecular components coexist in a single-vessel integrated reaction, the lack of physical partitioning poses inherent stability challenges, where residual unextended primers or oligos could act as competitive inhibitors during subsequent thermal cycles. To mitigate this risk, strict primer design rules were enforced to minimize 3’-end complementarity and maintain synchronized melting temperatures. Specifically, primer 3’-ends were rigorously screened to avoid sequences identical or highly homologous to the adapter-related motifs (including 5’-ACACTCTTT-3’and 5’-GTGACTGGAG-3’) to prevent mispriming. For scaling up to larger panels, the annealing time can be appropriately extended to 3 minutes to ensure balanced thermodynamic equilibration across complex primer pools. Furthermore, high-performance liquid chromatography (HPLC) purification is mandatorily required for all oligos to secure chemical purity. Beyond sequence variance, the molecular mechanism of OS-MPCR fundamentally depends on the integrity of the 5’-phosphate groups on the specific primers to drive splint-mediated assembly. Incomplete or failed 5’-phosphorylation during synthesis represents a recognized synthesis artifact that could potentially compromise amplification efficiency in real-world clinical application scenarios involving variable reagent quality. To evaluate the platform stability from a high-multiplexing perspective, we extended our testing using four independent panels scaled from 25 genes up to 207 genes. Encouragingly, no systemic reduction in target coverage or amplification failure was observed across these complex pools, which strongly supports the structural robustness of the multiplex assembly. Nevertheless, safeguarding oligonucleotide modification integrity remains a critical technical priority, and establishing rigorous raw material quality control guidelines (such as mass spectrometry verification for newly synthesized oligos) will be prioritized as a major focus in our future research and development phases to buffer against batch-to-batch chemical variations. Second, the current study is inherently limited by its retrospective design and relatively small cohort size for low-prevalence alterations, which may introduce selection bias toward high-abundance cases as previously noted. Large-scale, multicenter prospective trials are warranted in future studies to rigorously validate the clinical utility, robust workflow adaptability, and diagnostic accuracy of OS-MPCR in unselected real-world patient populations. Additionally, while our indexing prevents sample misassignment, it currently lacks the ability to correct for PCR duplicates or amplification errors. The introduction of Unique Molecular Identifiers (UMIs) is a planned future iteration that will enable consensus-based error correction, further enhancing sensitivity for ultra-low-frequency variants (<0.1%) required for advanced MRD applications [32]. Additionally, integrating the reverse transcription step directly into the one-step workflow will further streamline RNA-based fusion assays.

## Conclusions

In conclusion, we have evaluated a modified One-Step Multiplex PCR (OS-MPCR) strategy utilizing a splint-oligo design that offers a practical refinement over conventional two-step multiplex PCR library preparation workflows. By explicitly eliminating post-PCR transfer and handling steps, OS-MPCR establishes an intrinsic molecular barrier against amplicon carryover contamination in a closed-tube system. The assay demonstrates robust analytical sensitivity at low nucleic acid inputs (1 ng DNA, 5 ng RNA) and high diagnostic concordance with established hybrid capture methods in clinical FFPE lung cancer specimens, while significantly reducing manual hands-on time to approximately 20 minutes. Furthermore, the simplified single-vessel workflow reduces reagent overhead and operational complexity, making it an accessible alternative for targeted diagnostic testing. While these findings confirm OS-MPCR as a reliable and efficient option for targeted amplicon sequencing, larger multi-center cohorts remain necessary to further validate its clinical utility in diverse patient populations. Additionally, future technical iterations will incorporate Unique Molecular Identifiers (UMIs) to enhance error correction for ultra-low-frequency variant detection, while integrating direct reverse transcription to further streamline RNA-based fusion assays.

## Supporting information

S1 Table Detailed sequences of specific primers, indexing adapters and splint oligos used in OS-MPCR panels. (DOCX)

S2 Table Specific primers (GRCh37/hg19) of DNA panel. (DOCX)

S3 Table Specific primers (GRCh37/hg19) of RNA panel. (DOCX)

S4 Table Performance of DNA panel in pleural effusion samples. (DOCX)

S5 Table Performance of DNA panel in blood sample. (DOCX)

S1 Fig DNA Variant Concordance. (TIF)

S1 File Validation of Specificity and Technical Scalability of OS-MPCR Under High-Multiplexing Conditions. (DOCX)

S2 File Source Data for Figs 2–4, S1 Fig and Tables 3–4 (Excel format). (XLSX)
