# Supplementary material for: A novel one-step multiplex PCR method (OS-MPCR) for efficient NGS library preparation in cancer
Source: PLoS One. 2026 Sep 15;21(9):e0357776. doi: 10.1371/journal.pone.0357776 (PMC13577403; doi:10.1371/journal.pone.0357776)
Supplement: Supplementary file 1 [file pone.0357776.s001.docx]

**S1 Table. Detailed sequences of specific primers, indexing adapters and splint oligos used in OS-MPCR panels.**

| **Component** | **Full sequence (5’to 3’)** |
| --- | --- |
| Specific Primer F | 5’-P-CCCTACACGACGCTCTTCCGATCT-[Target-specific sequence]-3’ |
| Specific Primer R | 5’-P-TTCAGACGTGTGCTCTTCCGATCT-[Target-specific sequence]-3’ |
| Index i5 | 5’-AATGATACGGCGACCACCGAGATCTACAC-(i5 Barcode)-ACACTCTTT-3’ |
| Index i7 | 5’-CAAGCAGAAGACGGCATACGAGAT-(i7 Barcode)-GTGACTGGAG-3’ |
| Splint oligo 1 | 5’-GTCGTGTAGGGAAAGAGTGT-3’ |
| Splint oligo 2 | 5’-ACACGTCTGAACTCCAGTCAC-3’ |
