# Supplementary material for: A novel one-step multiplex PCR method (OS-MPCR) for efficient NGS library preparation in cancer
Source: PLoS One. 2026 Sep 15;21(9):e0357776. doi: 10.1371/journal.pone.0357776 (PMC13577403; doi:10.1371/journal.pone.0357776)
Supplement: Supplementary file 2 [file pone.0357776.s002.docx]

**S2 Table. Specific primers (GRCh37/hg19) of DNA panel.**

| Gene | Specific Primer F | Specific Primer R |
| --- | --- | --- |
| EGFR-L858 | 5’-P-CCCTACACGACGCTCTTCCGATCTGGAGGACCGTCGCTTGG-3’ | 5’-P-TTCAGACGTGTGCTCTTCCGATCTTGCATGGTATTCTTTCTCTTCCG-3’ |
| EGFR-19del | 5’-P-CCCTACACGACGCTCTTCCGATCTTGGATCCCAGAAGGTGAGAAAGT-3’ | 5’-P-TTCAGACGTGTGCTCTTCCGATCTGTGGGCCTGAGGTTCAGAGC-3’ |
| EGFR-T790 | 5’-P-CCCTACACGACGCTCTTCCGATCTGTGGACAACCCCCACGTGTG-3’ | 5’-P-TTCAGACGTGTGCTCTTCCGATCTGCACACACCAGTTGAGCAGG-3’ |
| EGFR-G719 | 5’-P-CCCTACACGACGCTCTTCCGATCTGCTTGTGGAGCCTCTTACACCC-3’ | 5’-P-TTCAGACGTGTGCTCTTCCGATCTCCTGTGCCAGGGACCTTACC-3’ |
| BRAF-V600 | 5’-P-CCCTACACGACGCTCTTCCGATCTGGCCAAAAATTTAATCAGTGGAAA-3’ | 5’-P-TTCAGACGTGTGCTCTTCCGATCTTTCATGAAGACCTCACAGTAAAA-3’ |
| ERBB2-20ins | 5’-P-CCCTACACGACGCTCTTCCGATCTGGTGTTTGGGGGTGTGTGGT-3’ | 5’-P-TTCAGACGTGTGCTCTTCCGATCTCACCAGCTGCACCGTGGATG-3’ |
| KRAS-G12 | 5’-P-CCCTACACGACGCTCTTCCGATCTACCTCTATTGTTGGATCATATTCGTCCA-3’ | 5’-P-TTCAGACGTGTGCTCTTCCGATCTAGGCCTGCTGAAAATGACTGAA-3’ |
| NRAS-G12 | 5’-P-CCCTACACGACGCTCTTCCGATCTGGGCTACCACTGGGCCTCA-3’ | 5’-P-TTCAGACGTGTGCTCTTCCGATCTGGTTTCCAACAGGTTCTTGCTGG-3’ |
| PIK3CA-E545 | 5’-P-CCCTACACGACGCTCTTCCGATCTAGAGTAACAGACTAGCTAGAGACAATGA-3’ | 5’-P-TTCAGACGTGTGCTCTTCCGATCTCCATTTTAGCACTTACCTGTGACTCCA-3’ |
