# Supplementary material for: A novel one-step multiplex PCR method (OS-MPCR) for efficient NGS library preparation in cancer
Source: PLoS One. 2026 Sep 15;21(9):e0357776. doi: 10.1371/journal.pone.0357776 (PMC13577403; doi:10.1371/journal.pone.0357776)
Supplement: Supplementary file 3 [file pone.0357776.s003.docx]

**S3 Table. Specific primers (GRCh37/hg19) of RNA panel.**

| Gene | Specific Primer F | Specific Primer R |
| --- | --- | --- |
| EML4(E2)-ALK(E20) | 5’-P-CCCTACACGACGCTCTTCCGATCTGGCTGATGTTTTGAGGCGTC-3’ | 5’-P-TTCAGACGTGTGCTCTTCCGATCTAGTAGTTGGGGTTGTAGTCGG-3’ |
| EML4(E6)-ALK(E20) | 5’-P-CCCTACACGACGCTCTTCCGATCTTCACCAGCTGAAAAGTCACAT-3’ | 5’-P-TTCAGACGTGTGCTCTTCCGATCTAGTAGTTGGGGTTGTAGTCGG-3’ |
| EML4(E13)-ALK(E20) | 5’-P-CCCTACACGACGCTCTTCCGATCTGGAGCAAAACTACTGTAGAGCCCA-3’ | 5’-P-TTCAGACGTGTGCTCTTCCGATCTAGTAGTTGGGGTTGTAGTCGG-3’ |
| EML4(E20)-ALK(E20) | 5’-P-CCCTACACGACGCTCTTCCGATCTCACACACCTTGACTGGTCCCC-3’ | 5’-P-TTCAGACGTGTGCTCTTCCGATCTAGTAGTTGGGGTTGTAGTCGG-3’ |
| CD74(E6)-ROS1(E34) | 5’-P-CCCTACACGACGCTCTTCCGATCTTGGATGCACCATTGGCTCCTG-3’ | 5’-P-TTCAGACGTGTGCTCTTCCGATCTAGTATGAAACTTGTTTCTGGTATCCA-3’ |
| KIF5B(E15)-RET(E12) | 5’-P-CCCTACACGACGCTCTTCCGATCTTGCAGAAATAGGAATTGCTGTGGGA-3’ | 5’-P-TTCAGACGTGTGCTCTTCCGATCTTGGTGTACCCTGCTCTGCCT-3’ |
| MET 14 exon skipping | 5’-P-CCCTACACGACGCTCTTCCGATCTAGATCAGAATTTCACAGGATTGATTGC-3’ | 5’-P-TTCAGACGTGTGCTCTTCCGATCTCTGTCAGAGGATACTGCACTTGTC-3’ |
