# Supplementary material for: A novel one-step multiplex PCR method (OS-MPCR) for efficient NGS library preparation in cancer
Source: PLoS One. 2026 Sep 15;21(9):e0357776. doi: 10.1371/journal.pone.0357776 (PMC13577403; doi:10.1371/journal.pone.0357776)
Supplement: Supplementary file 4 [file pone.0357776.s004.docx]

**S4 Table. Performance of DNA panel in pleural effusion samples.**

| **Dilution of EGFR T790M** | **Original (2.64%)** | **1%** | **0.5%** | **0.1%** |
| --- | --- | --- | --- | --- |
| OS-MPCR | 3.68% | 1.54% | 0.82% | 0.15% |
