# Supplementary material for: A novel one-step multiplex PCR method (OS-MPCR) for efficient NGS library preparation in cancer
Source: PLoS One. 2026 Sep 15;21(9):e0357776. doi: 10.1371/journal.pone.0357776 (PMC13577403; doi:10.1371/journal.pone.0357776)
Supplement: Supplementary file 5 [file pone.0357776.s005.docx]

S5 Table. Performance of DNA panel in blood sample.

|  | **blood sample 1** | **blood sample 2** | **blood sample 3** |
| --- | --- | --- | --- |
|  | **BRAF D594G** | **EGFR 19del** | **EGFR L858R** |
| OS-MPCR | 1.11% | 0.13% | 0.47% |
| Liquid-Phase Hybrid Capture | 0.63% | 0.29% | 0.42% |
