# Supplementary material for: A novel one-step multiplex PCR method (OS-MPCR) for efficient NGS library preparation in cancer
Source: PLoS One. 2026 Sep 15;21(9):e0357776. doi: 10.1371/journal.pone.0357776 (PMC13577403; doi:10.1371/journal.pone.0357776)
Supplement: Supplementary file 6 [file pone.0357776.s007.docx]

**S1 File. Validation of Specificity and Technical Scalability of OS-MPCR Under High-Multiplexing Conditions.**

**1. Experimental Rationale and Sample Description**

To objectively evaluate whether the single-vessel integration of the One-Step Multiplex PCR (OS-MPCR) assay introduces performance compromises (such as primer-primer dimers or severe off-target amplification) as the target scale expands, validation was conducted using four larger panels with increasing plexy.

Samples: Genomic DNA extracted from leukocyte samples (designated as Sample 1 to Sample 4) were utilized.

Input DNA Quantity: For all expanded testing panels was performed using a standardized input of 20ng total genomic DNA.

**2. Methods and Panel Specifications**

The physical design parameters of the four verification blocks are specified as follows:

Sample 1 (25-gene panel): Targeted panel featuring randomly designed primers.

Sample 2 (50-gene panel): Expanded target panel featuring randomly designed primers.

Sample 3 (100-gene panel): Medium-scale targeted screening panel featuring randomly designed primers.

Sample 4 (207-gene panel): Large-scale targeted profiling panel featuring randomly designed primers.

**Oligonucleotide Quality Control and Library Protocol**: All multiplex primers within the target pools were stringently purified using high-performance liquid chromatography (HPLC) to ensure high chemical purity. To balance enrichment kinetics across different multiplex primer densities, a stratified thermal profiling strategy was adopted during the PCR cycling stage: For the smaller panels (Samples 1 and 2; 25/50-gene panels), the annealing time was set to 90 seconds. For the larger panels (Samples 3 and 4; 100/207-gene panels), the annealing time was appropriately extended to 3 minutes.

This targeted extension allowed optimized thermodynamic equilibration across the dense, randomly designed primer networks, successfully suppressing transient, low-melting-point non-specific pairings. Following library preparation, the clean libraries were analyzed using capillary electrophoresis fragment analysis and sequenced on a high-throughput next-generation sequencing (NGS) platform.

**3. Comprehensive Performance Metrics across Large-Scale Panels**

The quantitative NGS mapping performance and enrichment quality attributes are compiled in Table S1.1. The microfluidic capillary electrophoresis fragment distribution profiles for each corresponding panel are illustrated in Fig S1.1.

Table S1.1. Sequencing Quality Control and Target Enrichment Metrics of OS-MPCR Across Four High-Multiplexing Scenarios.

| **Sample** | **Panel** | **Mapped Data (Mb)** | **Mapping rate (%)** | **On-target rate (%)** | **On-target rate in mapped reads (%)** | **Primer-Dimer Ratio (%)** |
| --- | --- | --- | --- | --- | --- | --- |
| Sample 1 | 25-gene | 425.06 | 99.31% | 98.73% | 99.92% | 3.8% |
| Sample 2 | 50-gene | 587.38 | 99.65% | 99.32% | 99.93% | 2.6% |
| Sample 3 | 100-gene | 711.85 | 99.83% | 99.65% | 99.94% | 0.6% |
| Sample 4 | 207-gene | 783.88 | 99.90% | 99.46% | 99.62% | 0.3% |

**Fig S1.1. Capillary Electrophoresis Fragment Analysis Matrix of Expanded High-Multiplexing Panels.**
